# Partial reconstruction of the external ear after a trauma-simple and efficient techniques

**DOI:** 10.1590/S1808-86942010000100002

**Published:** 2015-10-17

**Authors:** Monica Rebello Ottat

**Affiliations:** 1 Full Member of the Brazilian Society of Plastic Surgery. MSc. Student in Plastic Surgery - Graduate Program - UFRJ and teaching supervisor - Plastic and Repair Surgery Department - Hospital Municipal Barata Ribeiro - RJ

**Keywords:** traumatic amputation, surgery, acquired ear deformities, pinna

## Abstract

The growing number of ear trauma cases has brought about the need for simple and efficient solutions.

**Aim:**

To be able to rebuild an ear partially injured by trauma, even in a small hospital setting. Many techniques can be used; however, the solution can be the use of cutaneous or chondrocutaneous flaps.

**Methods:**

To reconstruct the ear upper or middle thirds we used a mastoid cutaneous flap with or without a cartilage graft or a chondrocutaneous flap - chosen considering the size of the defect and the need for a cartilage graft.

**Results:**

The patients were able to wear glasses and were pleased with the results. Most of the patients ended up having a good helix and cartilaginous contour. Some had hypertrophic scars on the grafted areas and two required a second procedure.

**Conclusion:**

Mastoid cutaneous flaps or local chondrocutaneous flaps can represent a good solution for partial ear loss after trauma. Most of the patients had very high expectations concerning the procedure. This poses a major technical challenge for the surgeon and requires prior discussion with the patients regarding the true possibilities of outcome.

## INTRODUCTION

The Incidence of partial ear deformities stemming from the most varied etiologies (human bites, animal bites, fights, sports, car accidents, infections, burns, etc.) has been on the rise in our society^1^, ^2^, ^3^, ^4^. With such problem in mind, we hereby present two possible techniques which are easy to be performed in humbler hospital settings.

When these regions can not be treated by approximating the wound borders, there are numerous techniques for partial reconstruction of the pinna and many of them are rather complex.

The possibility of doing these procedures under local anesthesia, caring for elderly or very young patients who do not wish to be submitted to a removal of costal cartilage, and to adjust the technique to the characteristics of our hospital – which has fewer resources, showed that the skin flap advocated by Dieffenbach in 1845^5^, with the association of retroauricular skin and/or Antia and Buch's chondrocutaneous flap from 1967^6^, provides very satisfactory functional and cosmetic results.

The goal of the present paper is to show the surgeons who work in emergency settings that we can develop an ear concha framework and a very reasonable cosmetic contour to patients using established techniques which are simple and achieving similar results to those obtained from more complex techniques which bear higher morbidity rates.

## PATIENTS AND METHODS

This project was assessed and approved by the Ethics and Research Committee (131/08).

In order to be included in the protocol, the patients had to have had a partial loss of the pinna, involving the upper or middle third, stemming from recent or late trauma.

Patients with total loss or those who lost only the ear lobe were not included.

The patients (n=19) were operated in Rio de Janeiro, between 2000 and 2008.

The choice of technique was based on lesion size, remaining tissue^7^,^8^ and whether or not the patient had lost cartilaginous framework.

In cases of defects with loss of cartilaginous framework of more than 2.5cm, we chose the rectangular skin flap (Figures 1a, 1b) – in the same width of the defect - with its base on the mastoid region, ipsilateral to the lesion and which ends on its border (16 cases). The incision is made on the defect's border and the skin detachment continues on the retroauricular skin^9^ until reaching the hair line, going through the mastoid (Figures 2a, 2b, 2b2, 2c, 2d). One important detail is to anchor the pinna to the mastoid with a 4.0 mono nylon wire, in order to reduce flap tension. Here one can assess the possibility of ipsi or contralateral pinna cartilage harvesting, this framework is grafted to the defect area, anchoring it with 5.0 mono nylon wires and covering it with the flap (Figures 5a, 5b). The skin suture is done with 5.0 mono nylon wire, except for the distal end of the flap, where we use 6.0 mono nylon wire. On a second look, 3 weeks later, the flap is released, its base is cut and the flap is moved towards the ear in order to make the retroauricular groove; the anchoring points are removed and the flap donor area is grafted (with contralateral retroauricular or supraclavicular skin). We dress it with cotton soaked in saline solution on the ear curves, we protect the area and the contralateral ear with gauze and padded dressing - we use bandage around the patient's head in order to make the “hat”. In one case there was no need to graft cartilage, or to release the flap, since the 3cm loss involved only the helix border.

**Figure 1a:**
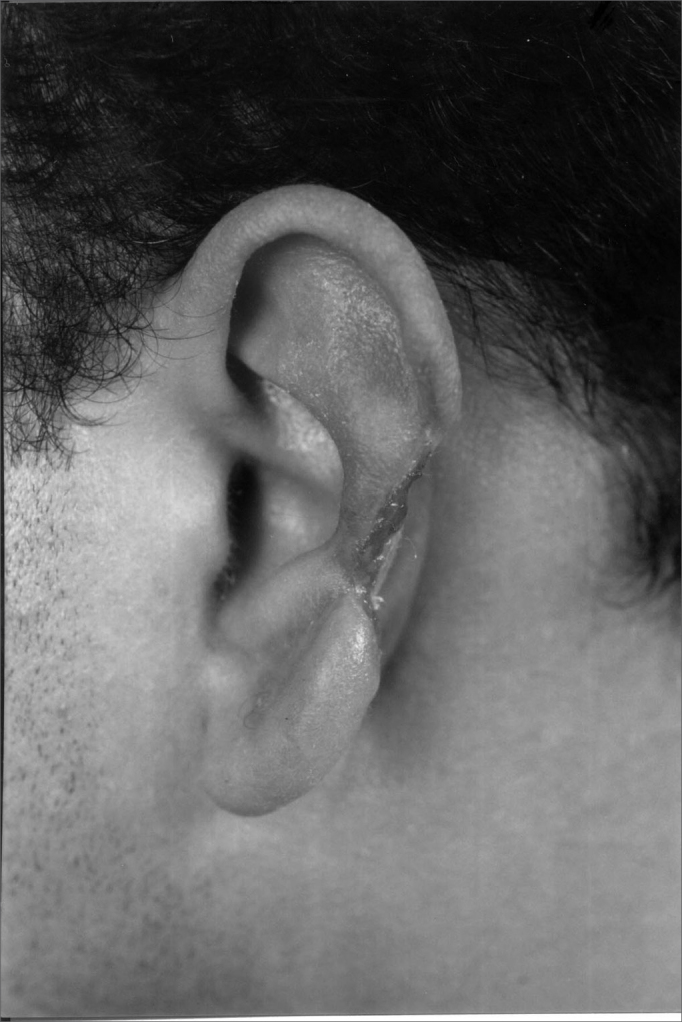
Man suffered human bite.

**Figure 1b:**
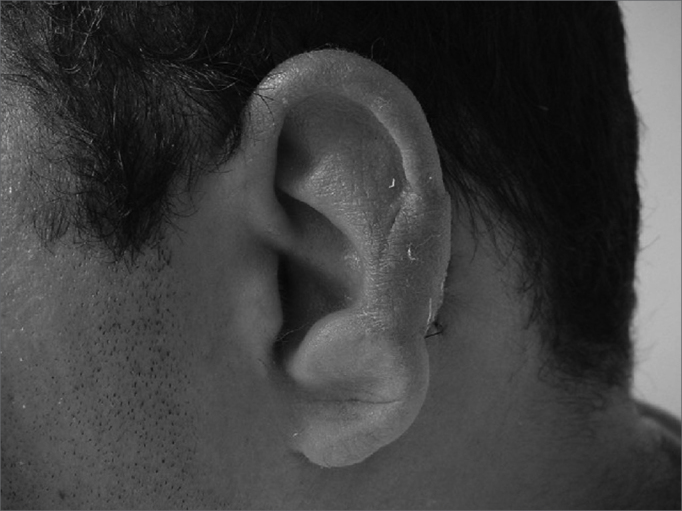
Reconstruction with mastoid skin flap associated with the contralateral pinna cartilage graft. 7 day post-op.

**Figure 2a:**
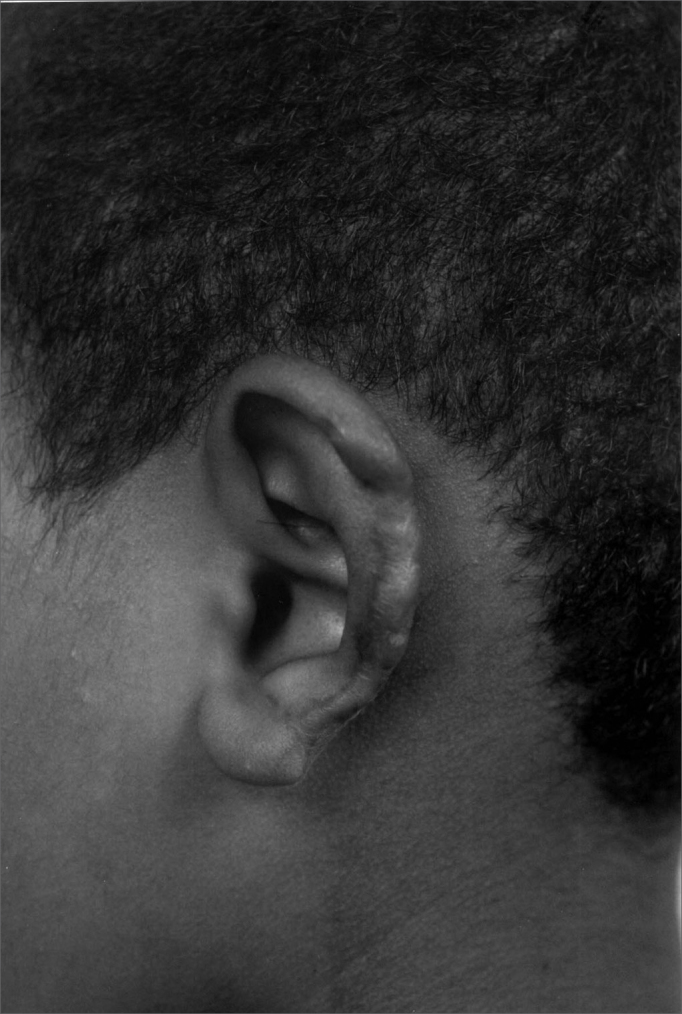
Child bit by a dog.

**Figure 2b:**
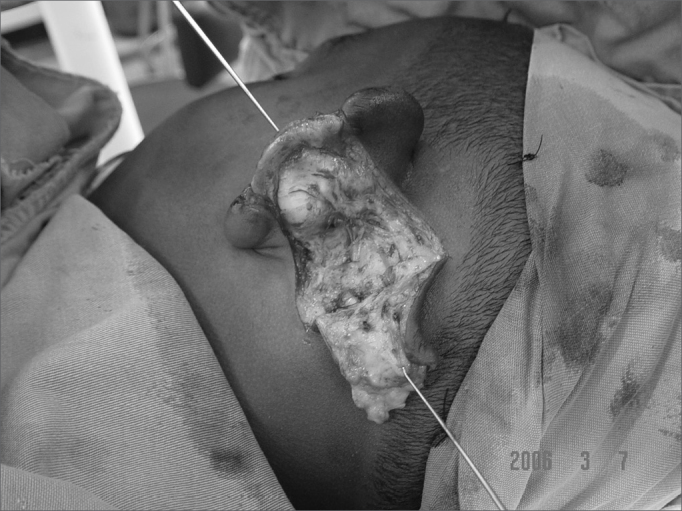
Mastoid skin flap without cartilage graft.

**Figure 2b2:**
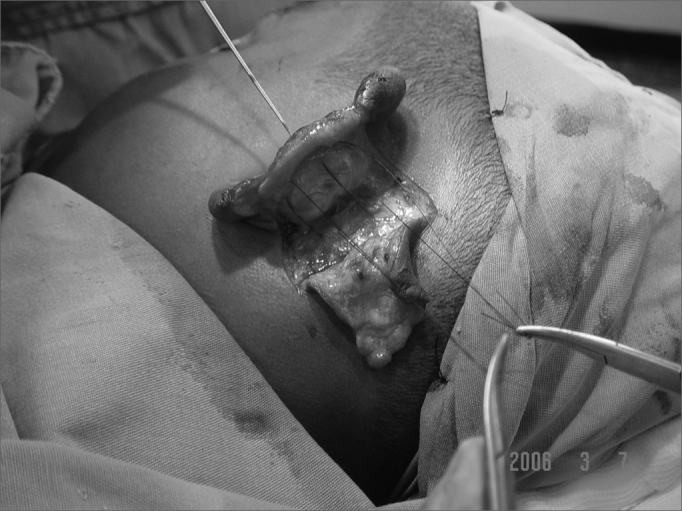
Mastoid concha fixation in order to reduce the skin flap when advanced.

**Figure 2c:**
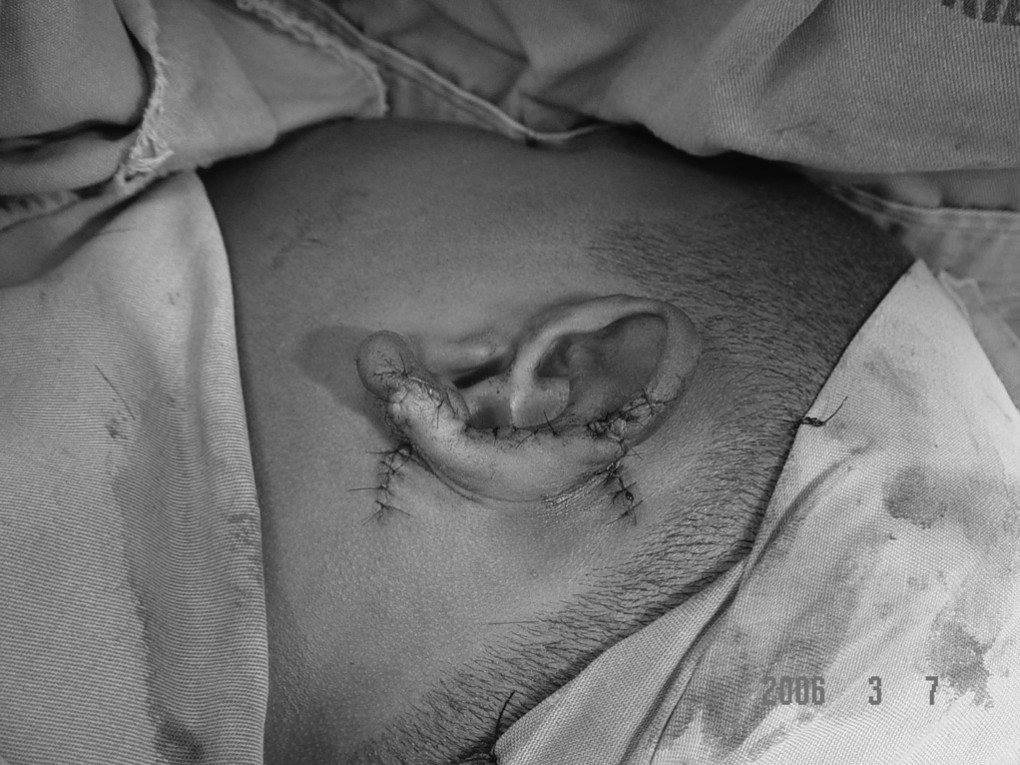
Flap advancement.

**Figure 2d:**
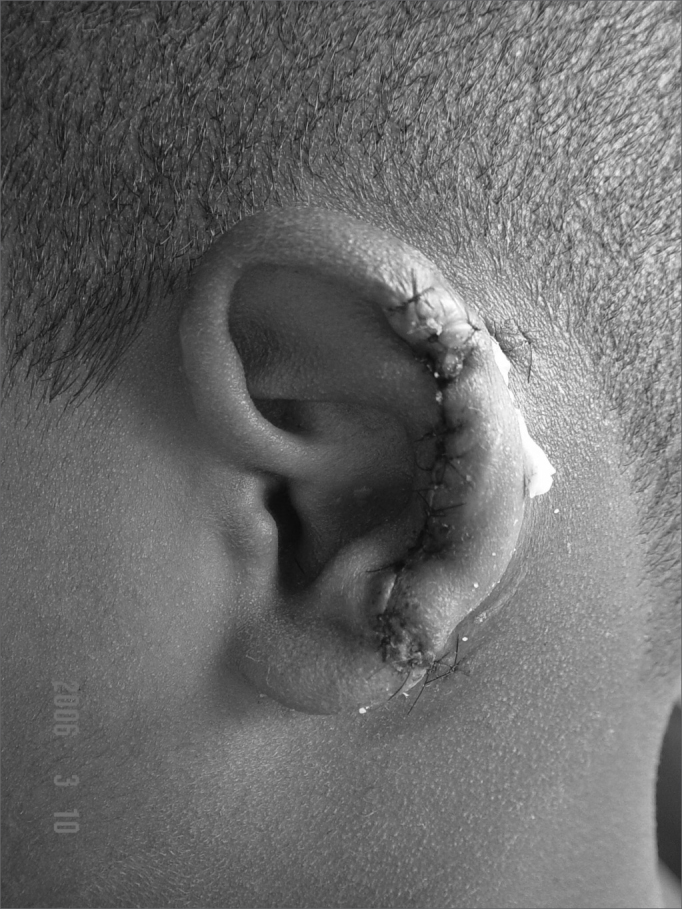
Immediate post-op.

**Figure 5a:**
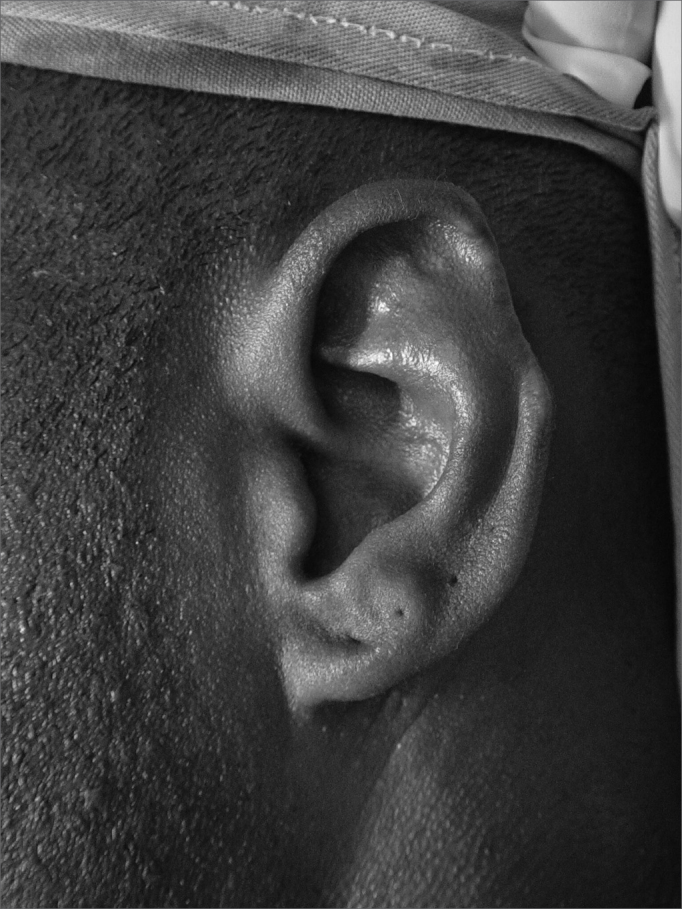
Pre-op. Compliance weapon wound.

**Figure 5b:**
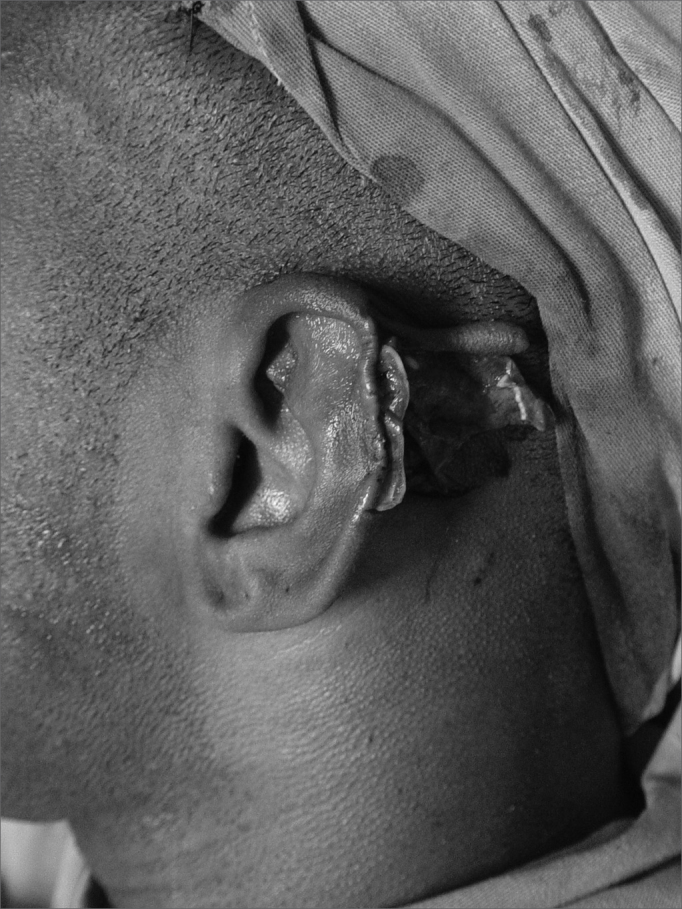
Ipsilateral pinna graft with an elevated skin graft.

In cases of defects smaller than 2.5cm (3 cases) and where the cartilage loss happened only on the helix border we used a superoinferior chondrocutaneous flap, as described by Antia and Buch6 (Figures 3a, 3b). One incision is made on the border between the scapha and the helix, parallel to the defect, encompassing the skin and the cartilage, without compromising the posterior ear skin - which will be used as flap base. We perform a medial detachment under the pinna cartilage in order to enhance flap advancement until they cover the defect and are sutured with 5.0 mono nylon wires (joining the helix cartilage) and 6.0 mono nylon for skin closure. We also use cotton molds and “hat-type” dressing.

**Figure 3a:**
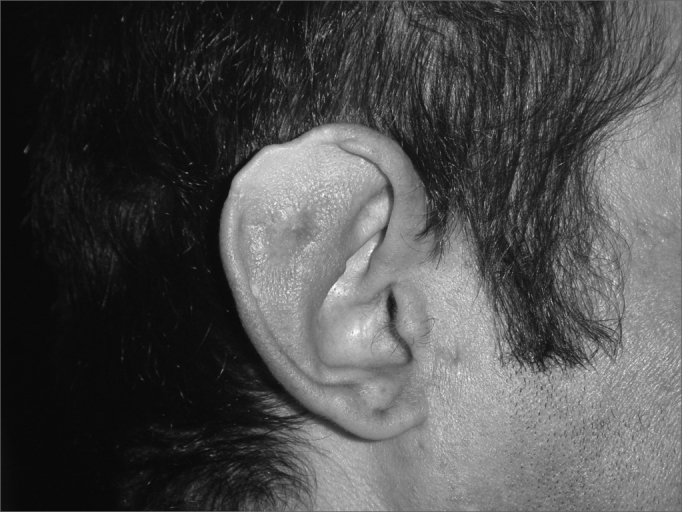
Post-piercing chondritis.

**Figure 3b:**
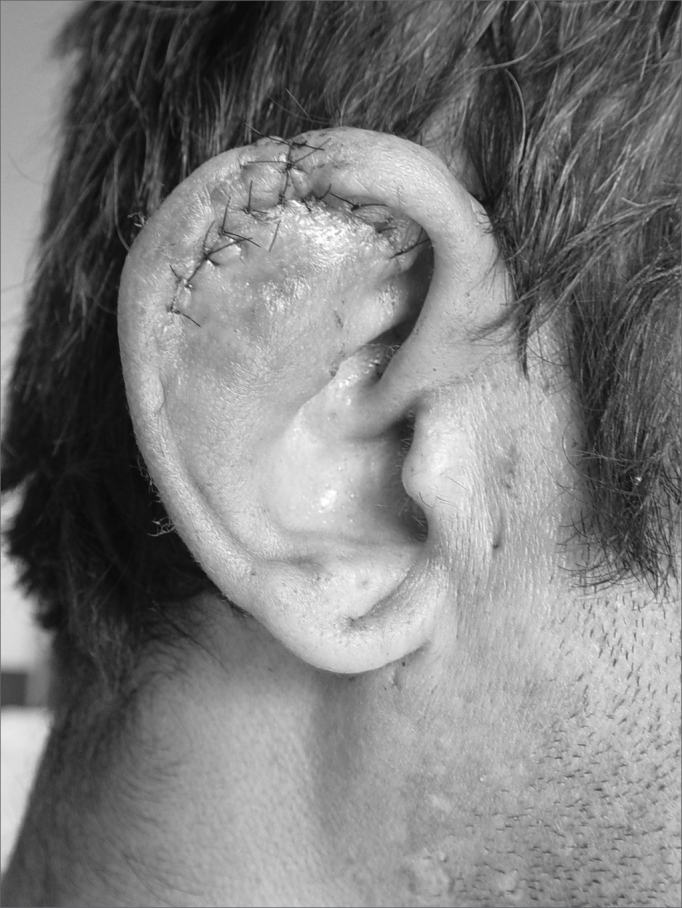
Two-day post-op of inferior and superior condrocutaneous flap.

## RESULTS

We treated 19 patients with ages varying between 7 and 60 years, 15 men and 4 women.

The defects varied in size between 1.5 cm and 3.5 cm and they were located on the upper or middle third of the ear. The trauma etiology varied, 10 patients suffered human bites, three had had infections (piercing and scabies), three were injured by non-lethal weapon, one by car accident, one by dog bite and one suffered a burn.

**Graph 1:**
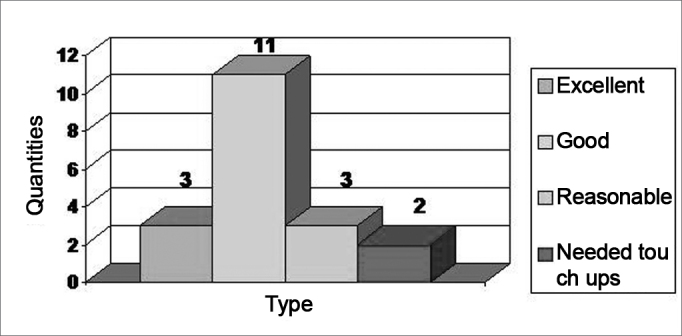
Cosmetic Results Obtained (19 cases).

The technique chose varied according to defect size and the need for a cartilaginous graft (Graph 1). We used the skin graft in 16 cases; for one of them it was not necessary to graft cartilage; in three other patients we used the chondrocutaneous flap; and in two of the latter the two approaches were used in association (Figures 4a, 4b).

**Figure 4a:**
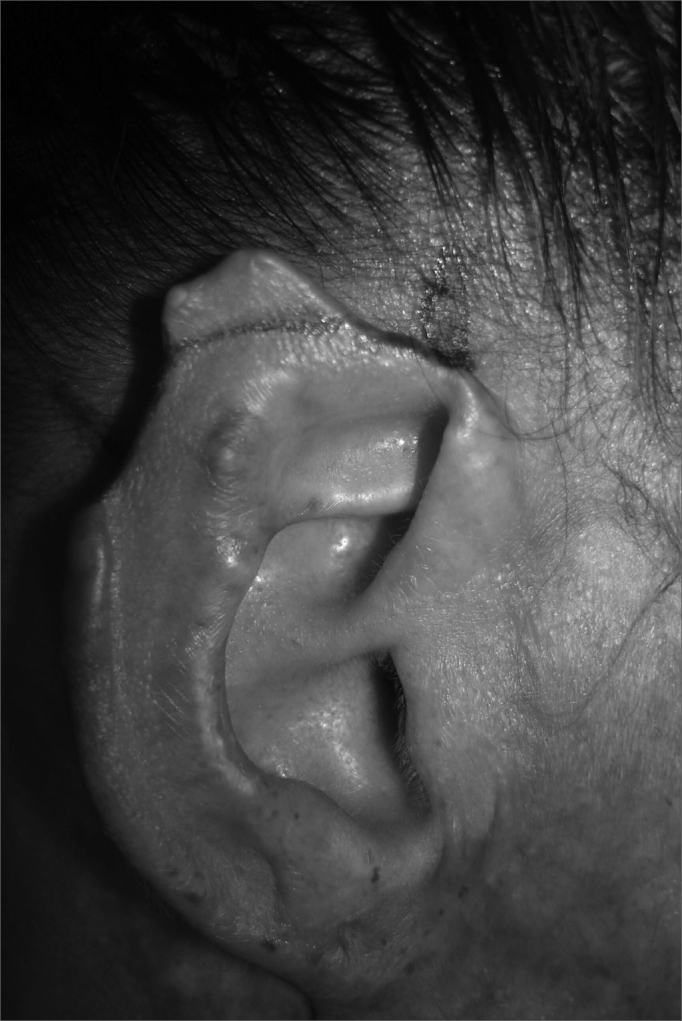
Burn.

**Figure 4b:**
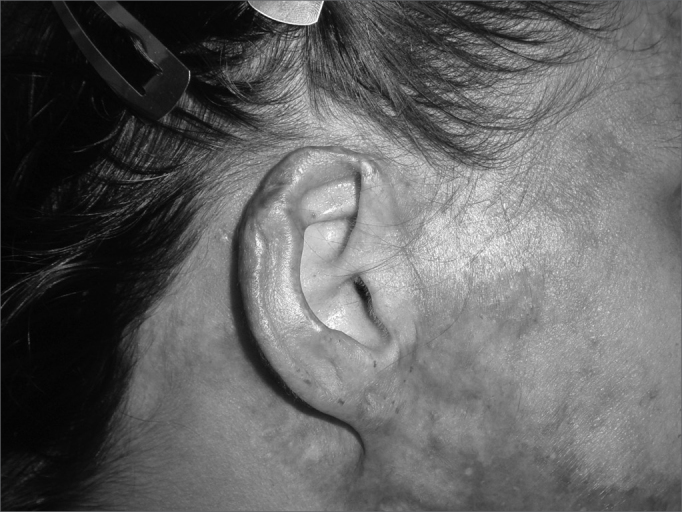
Skin flap with contralateral cartilage graft and 2 months after a second procedure with inferior condrocutaneous flap. Three-month final post-op.

**Graph 2:**
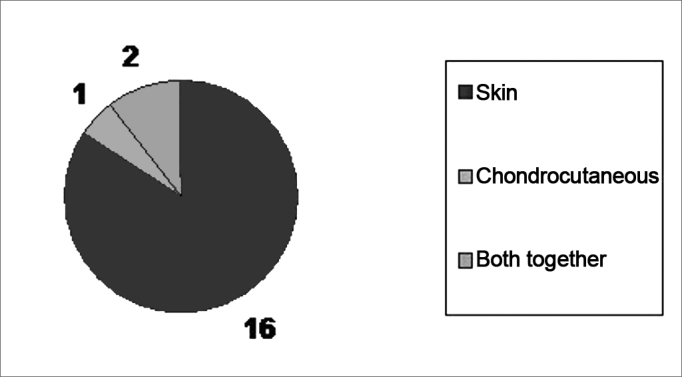
Techniques Used (19 cases).

The results were considered satisfactory by all the patients, including the functional aspect (being able to wear glasses) and the team considered 3 results as excellent, 11 results were deemed good, three flap donor areas which received skin grafts evolved with hypertrophic scars and two required late touch ups (after 2 years) (Graph 2).

Results were satisfactory for the patients and for the medical team. We believe the techniques proposed helped the patients functionally and cosmetically.

The physician-patient relationship is extremely important in order to establish real result possibilities, since these patients tend to expect a repair that would give them back the same ear they had before the trauma. It was possible to treat children and elderly patients who were seeking a simpler treatment, with fewer possibilities for complications, and one which would return them as soon as possible to their activities.

## DISCUSSION

We identified human bite as the main cause of facial loss for trauma and we noticed that the incidence of this problem is on the rise in our country. Many are the techniques^10^, ^11^, ^12^, ^13^, ^14^, ^15^ proposed to rebuild segments of the ear pinna, and this shows that we are still short of the ideal approach, even because the lesions vary in size, shape and left over tissue.

We believe that despite the numerous existing approaches^5^,^6^, some already known and established can help. We chose approaches which can be carried out under local anesthesia and in humbler hospital facilities - not needing fancy equipment.

**Figure 5c:**
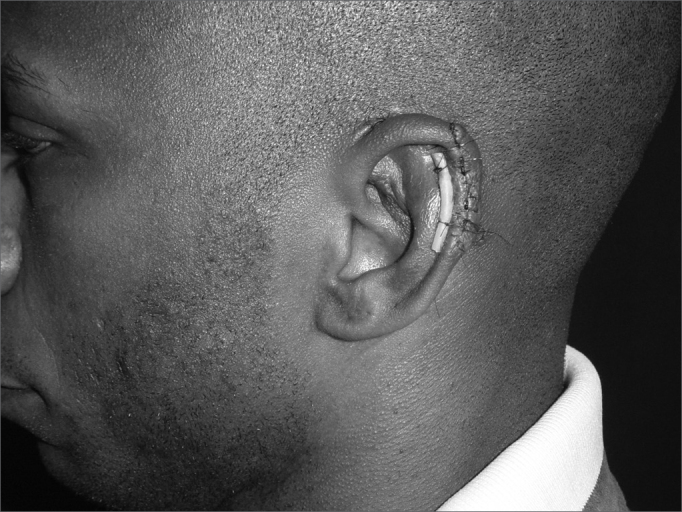
Second day of post-op.

As it happens to many authors^13^,^16^, we believe the chondrocutaneous flap is the best choice, as long as the loss is equal to or smaller than 2.5 cm. Since it is a flap which can be advanced superiorly, inferiorly or in both directions, and for being a composite flap, the reconstruction can be completed in one single procedure, without damaging the donor area, and it yields very good cosmetic results.

The skin flap is well indicated in cases of lesions greater than 2.5cm, and where there was a loss of cartilage framework. Nonetheless, one more procedure was needed: there was a flap donor area and another one for the skin graft used to cover it and, in some cases, we noticed that the grafted area presented non-cosmetic scars. The authors describe this need for more than one surgical procedure and touch ups in the flap donor area.

The use of contralateral^16^ and homolateral^13^ pinna cartilage graft was a good choice. The results were very satisfactory and without complications. Such choice was based on papers from authors like Rieck^17^ in 2005, Bro-dland^18^, also in 2005, Sclafan^19^, in 2006, and Ohara^20^, in 1997, who showed complications such as pleura perforations, chest wall deformities and chest scoliosis, amount others, caused by the removal of costal cartilage and our very characteristic of a small hospital setting with scarce resources.

## CONCLUSION

The choice of easy techniques to rebuild the partial ear loss caused by trauma is hereby presented with the goal of helping surgeons who face this increasingly frequent problem and who work in hospitals with less material resources.

We have concluded that the use of skin flaps on the mastoid, associated or not with homo or contralateral cartilage graft to fix defects larger than 2.5cm and chondrocutaneous flaps from the helix border ipsilateral to the defect, done in one single procedure for lesions smaller than 2.5cm - which allow for partial reconstruction of the pinna in order to obtain a cartilaginous framework and good skin cover, not presenting complications, showed results very similar to those achieved with more complex techniques, and those causing greater morbidities proposed in the literature.

## References

[1] Crikelair GF (1956). A method of partial ear reconstruction for avulsion of the upper portion of the ear. Plast Reconstr Surg..

[2] Giraldo-Anisio F, Garcia-Dominguez MD, Abad-Mariñez A. (1997). One-stage immediate reconstruction of partial auricular amputation by a dog bite. Int J Oral Maxillofac Surg..

[3] Godwin Y, Allison K., Waters R. (1999). Reconstruction of a large defect of the ear using a composite graft following a human bite injury. Br J Plast Surg..

[4] Margulis A (2003). Ear reconstruction after auricular chondritis secondary to ear piercing. Plast Reconstr Surg..

[5] Dieffenbach JF (1845). Die Operative Chirurgie.

[6] Antia NH, Buch VI (1967). Chondrocutaneous advancement flap for marginal defect of the ear. Plast Reconstr Surg..

[7] Brent B. (1977). The acquired auricular deformity. A systematic approach to its analysis and reconstruction. Plast Reconstr Surg..

[8] Musgrave RH, Garrett WS (1967). Management of avulsion injuries of external ear. Plast Reconstr Surg..

[9] Lewin ML (1950). Formation of the helix with a postauricular flap. Plast Reconstr Surg..

[10] Avelar JM (1997). Creation of the auricle.

[11] Chana JS, Grobbelaar, Gault DT (1997). Tissue expansion as an adjunct to reconstruction of congenital and acquired auricular deformities. Br J Plast Surg..

[12] Cronin TD (1952). One stage reconstruction of the helix: two improved methods. Plast Reconstr Surg..

[13] Millard DR (1966). The chondrocutaneous flap in partial auricular repair. Plast Reconstr Surg..

[14] Quaba A. (1988). Reconstruction of a posttraumatic ear defect using tissue expansion: 30 year after Neumann. Plast Reconstr Surg..

[15] Rieck B. (2005). Reconstruction of the external ear after trauma or tumor surgery. Eur J Plast Surg..

[16] Steffensen WH (1984). Comments on reconstruction of the external ear. Plast Reconstr Surg..

[17] Rieck B. (2005). Reconstruction of the external ear afetr trauma or tumor surgery. Eur J Plast Surg..

[18] Brodland D. (2005). Auricular Reconstruction. Dermatol Clin.

[19] Sclafani AP, Mashkevich G. (2006). Aesthetic Reconstructionof the Auricle. Facial Plast Surg Clin N Am.

[20] Ohara K, Nakamura K, Ohta E. (1997). Chest Wall deformities and thoracic scoliosis after costal cartilage graft harvesting. Plast Reconstr Surg..

